# Quantification of liver fat fraction using T1-weighted mDixon MRI in young patients with ataxia telangiectasia undergoing whole-body MRI: an exploratory study

**DOI:** 10.1186/s13023-025-03786-1

**Published:** 2025-06-21

**Authors:** Soma Kumasaka, Rafal Panek, Renata Neves, Mohnish Suri, Sumit Jagani, Yuka Kumasaka, Yoshito Tsushima, Robert A. Dineen

**Affiliations:** 1 Radiological Sciences, Mental Health and Clinical Neuroscience, School of Medicine, University of Nottingham, Nottingham, UK; 2 Department of Diagnostic Radiology and Nuclear Medicine, Gunma University Graduate School of Medicine, Maebashi, Gunma Japan; 3 Medical Physics and Clinical Engineering, Nottingham University Hospitals NHS Trust, Nottingham, UK; 4 Department of Radiology, Nottingham University Hospitals NHS Trust, Nottingham, UK; 5 Nottingham Clinical Genetics Service, Nottingham University Hospitals NHS Trust, Nottingham, UK; 6 Sir Peter Mansfield Imaging Centre, University of Nottingham, Nottingham, UK; 7 NIHR Nottingham Biomedical Research Centre, Nottingham, UK; 8 Room A39, Precision Imaging, Medical School, Queen’s Medical Centre, Nottingham, NG7 2UH UK

**Keywords:** Ataxia–telangiectasia (A–T), Steatosis, Fat fraction, MRI, Dixon

## Abstract

**Background:**

Ataxia–telangiectasia (A–T) is an inherited multiorgan disorder with onset in childhood. Liver involvement, with steatosis and subsequent fibrosis, is increasingly recognized in children and young people with A–T.

**Purpose:**

To evaluate feasibility of T1-weighted two-point mDixon MRI for identification of liver steatosis in children with A–T and conduct exploratory analysis of relationships between MRI-quantified liver fat fraction and clinical and laboratory measures.

**Study type:**

Post hoc analysis of prospectively collected research data.

**Population:**

16 participants (8 female) with A–T aged 4.8–16.6 years.

**Field strength/sequence:**

3.0-T, two-point T1-weighted mDixon.

**Assessment:**

Participants underwent whole-body MRI including T1-weighted mDixon. Water/fat signal percentage images were generated. Hepatic T1 fat fraction (T1-FF) was calculated from regions-of-interest placed in the right anterior, right posterior and left hepatic lobes. T1-FF > 5.56% was used as the diagnostic criterion for hepatic steatosis.

**Statistical tests:**

Group comparisons of variables between participants with and without previous diagnosis of liver steatosis were made using independent sample Mann–Whitney U. Associations between T1-FF and age, neurological severity and of liver function tests were tested with Spearman correlation. Statistical significance was pre-specified as p < 0.05.

**Results:**

Analyzable T1-weighted mDixon data was available for 11 participants. Five MRI datasets were discarded due to motion artefact (n = 3) or incorrect archiving of the original water image (n = 2). Median liver T1-FF was 11.3% (4.7–49.7%), and 10/11 (91%) of participants had evidence of steatosis. Participants with previous diagnosis of steatosis had higher T1-FF than those without (median 32.7% [9.7–49.7%], versus 10.3% [4.7–11.3%], *p* = 0.030). T1-FF correlated most strongly with alanine transaminase (r = 0.76, *p* = 0.007) and γ-glutamyltransferase (r = 0.76, *p* = 0.006).

**Conclusion:**

T1-weighted mDixon MRI is feasible for detecting steatosis in children with A–T, although motion artefacts reduced data completeness. MRI-quantified liver T1-FF correlates with markers of liver health. We found higher prevalence of liver steatosis using T1-weighted mDIXON than previously reported in pediatric A–T cohorts.

## Introduction

Ataxia–telangiectasia (A–T) is an autosomal recessive disorder that is characterized by several distinct clinical features, including progressive cerebellar neurodegeneration, ocular telangiectasia, immunodeficiency, cancer susceptibility and radiation sensitivity [1–3]. A–T is caused by pathogenic or likely pathogenic variants in the ataxia–telangiectasia mutated (ATM) gene, which results in a complete deficiency or dysfunction of the ATM protein kinase, a key activator of the cellular responses to double-stranded DNA breaks and oxidative stress [2, 4–6].

Recent studies have shown that liver disease is common in people with A–T, manifest as elevated liver enzymes and hepatic steatosis on ultrasound. The liver disease in A–T emerges in the second decade, being present in around 40% of children and young adults overall, rising to 60% in people over 12 years and 90% in people over 20 years [7, 8]. Hepatopathy in A–T is typically mild and does not result in impaired synthesis or detoxification function of the liver. However, several case reports have documented the occurrence of severe conditions, including liver failure, liver steatosis, liver cirrhosis, and hepatocellular carcinoma (HCC) in patients with A–T [8, 9].

Currently, hepatic needle biopsy is considered the gold standard for diagnosing liver steatosis [10]. However, biopsy is an invasive technique with the inherent potential to lead to an inaccurate diagnosis due to the possibility that the sample obtained may not be representative of the entire liver [11]. Given these considerations, it has been proposed that liver imaging should be employed as a non-invasive alternative to histological assessment. This is a particularly promising approach for patients with A–T, where liver biopsy is typically a challenging procedure [12, 13]. Ultrasound is widely used as a non-invasive imaging modality for detecting liver steatosis, which is seen as increased liver parenchymal echogenicity. Although a method of quantitative evaluation of liver fat using ultrasound has emerged in recent years, this method does not directly measure fat; rather, it utilizes the attenuation of ultrasonic waves due to fat [14, 15]. Transient elastography is a non-invasive method for estimating liver steatosis and fibrosis, providing a parameter that correlates with (rather than directly measuring) liver fat fraction. Both ultrasound and transient elastography have been used previously to detect liver involvement in A–T [12, 13].

Magnetic resonance imaging (MRI) allows non-invasive liver fat quantification with the distinct advantages of comprehensive hepatic coverage during acquisition. In order to optimize the wide spatial coverage of MRI, several techniques have been developed to quantify hepatic fat, based on the different physical and chemical properties of water and fat protons. The chemical shift-based water–fat separation method (so-called the Dixon method) is the most widely used among these methods to date. This technique is founded upon the different resonance frequencies between protons bound in a fat and water molecule [16, 17].

To date, no studies have been published that have used the Dixon method for the evaluation of liver steatosis in patients with A–T. Furthermore, children with A–T experience involuntary movements and breathing difficulties related to respiratory problems that may interfere with successful image acquisition, and there have been no reports on whether the Dixon method can be applied to assess hepatic steatosis in these patients. This study aimed to assess the feasibility of the Dixon method for the evaluation of liver steatosis in children with A–T.

## Materials and methods

This study is a post hoc analysis of whole-body T1-weighted modified Dixon (mDIXON) images from participants prospectively recruited to a feasibility study of whole-body MRI for cancer screening in children and young people with A–T. Full details of the study rationale, design, eligibility criteria, recruitment and MRI protocol have been published previously [18]. The study was approved by the UK Health Research Authority ref. 22/YH/0053 and was conducted in accordance with the ethical principles of the Declaration of Helsinki. Informed consent was obtained from the parent of participants aged under 16 years, or from the participant directly if aged 16–18 years.

### Participants

Participants were recruited through the National Pediatric A–T Clinic at the Nottingham University Hospitals NHS Trust between October 2022 to September 2023. Inclusion criteria for the study were as follows: (1) confirmed diagnosis of A–T by genetic testing confirming biallelic *ATM* pathogenic or likely pathogenic variants and assay confirming non-functioning or hypo-functioning ATM protein kinase; (2) aged 4–18 years; (3) able to undergo MRI scan without sedation or general anesthetic, after age-appropriate preparation, and (4) able to give informed consent (if aged 16 years and older), or have a parent is able to give informed consent. Participants who completed the whole body T1-weighted mDIXON scan are reported in the analysis below.

Clinical data were obtained from the medical records from the date closest to the MRI, including laboratory test results (α-fetoprotein [AFP], Alkaline phosphatase [ALP], Alanine transaminase [ALT], γ-glutamyl transferase [GGT], Total bilirubin [T-Bil], Albumin, hemoglobin A1c [HbA1c]), height, weight and use of tube feeding, and A–T subtype (classical or variant).

For the purpose of nutritional status classification, the body mass index (BMI) was calculated from the height and weight and expressed as the BMI z-score relative to the normative values for age and sex using data and formula provided by the World Health Organization [19]. The A–T Neurological Examination Scale Toolkit (A–TNEST) was utilized to evaluate the severity of neurological dysfunction in all patients [20]. This provides a score out of 100, with lower scores indicating more severe neurological dysfunction.

### MRI protocol

As these scans were performed for research purposes, scans were performed without sedation or anaesthesia in all cases. In preparation for the MRI scan, participants and their families were sent an age-appropriate information sheet about the procedure and a link to an online animation [21], and on the day of the scan underwent play preparation by an experienced paediatric MRI radiographer using an MRI simulator or Philips ‘Kitten’ scanner [22] as appropriate to their needs. No specific fasting or dietary instructions were given. The imaging of all participants was conducted on a 3-Tesla MRI system (Elition, Philips Medical Systems, Eindhoven, The Netherlands) using a 32-channel torso coil. The axial T1-weighted two-point mDixon protocol comprised the following parameters: two TEs with an initial TE of 1.34 ms and a delta TE of 2.6 ms; a repetition time (TR) of 4.1 ms; and a flip angle (FA) of 15°. The field of view (FOV) was 550 × 299 × 250 mm, the matrix size 368 × 200, the acquired voxel size 1.5 × 1.5 × 4 mm, and the acquisition time 26 s per station. The images were processed using commercially available software (Philips Advanced Visualization Workspace) to generate water/fat signal percentage maps [23]. Due to the respiratory problems experienced by children with A–T, participants were not asked to breath-hold during the scan, but instead continued with normal resting ventilation.

### Image analysis

**Fig. 1:**
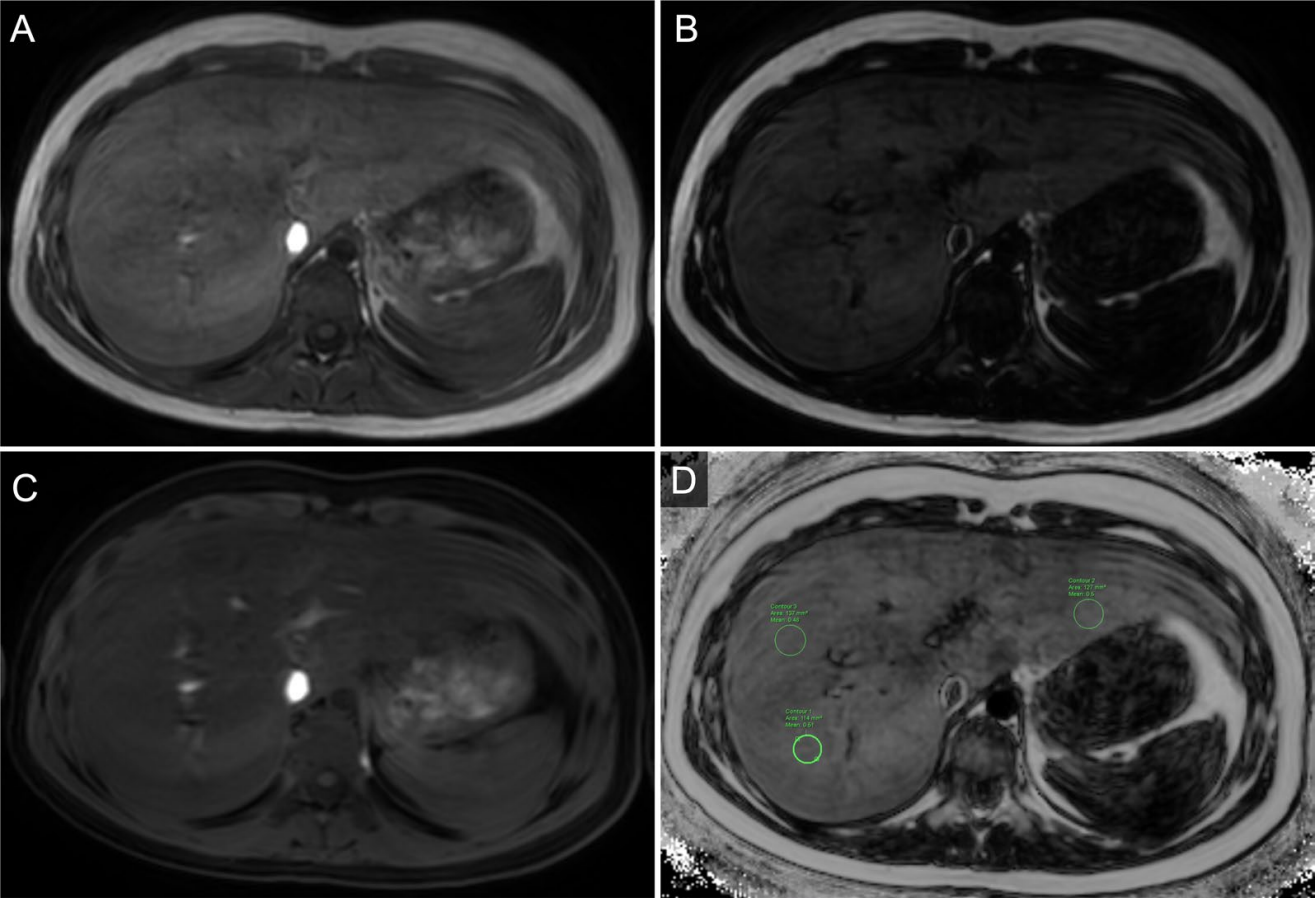
Example of **A** in-phase, **B** fat-image, **C** water-image and **D** T1 weighted mDixon-based fat fraction image with ROIs placement of the liver from a 15-year-old participant with A–T

The MRI images were analyzed by a radiologist (with more than 10 years of clinical experience in MRI interpretation) who was blinded to the clinical profiles. Three regions of interest (ROIs), each exceeding 1 cm^2^, were placed on the left hepatic lobe and anterior and posterior segments of the right hepatic lobe on the water/fat signal percentage map, excluding blood vessels, bile ducts, and the gall bladder. The mean value, which has been commonly used for assessing liver steatosis using the Dixon method in recent studies, was adopted as the T1-weighted mDixon-based fat fraction value (T1-FF) [17, 24, 25] (Fig. 1).

As the exact threshold for steatosis is not fully understood, we adopted a commonly used fat-fraction threshold of 5.56% to distinguish steatosis [23, 26].

### Statistical analysis

Data are presented as median (range). Group comparisons are made using independent sample Mann–Whitney U test. Spearman’s correlation coefficients were calculated to describe the associations between the T1-FF, age, A–TNEST, and laboratory data. The significance threshold was specified as *p* < 0.05. As this is an exploratory study multiple test correction was not applied [27]. All statistical analyses were performed using commercially available software (SPSS Statistics, version 29; IBM Corp., Armonk, NY, USA).

## Results

**Fig. 2:**
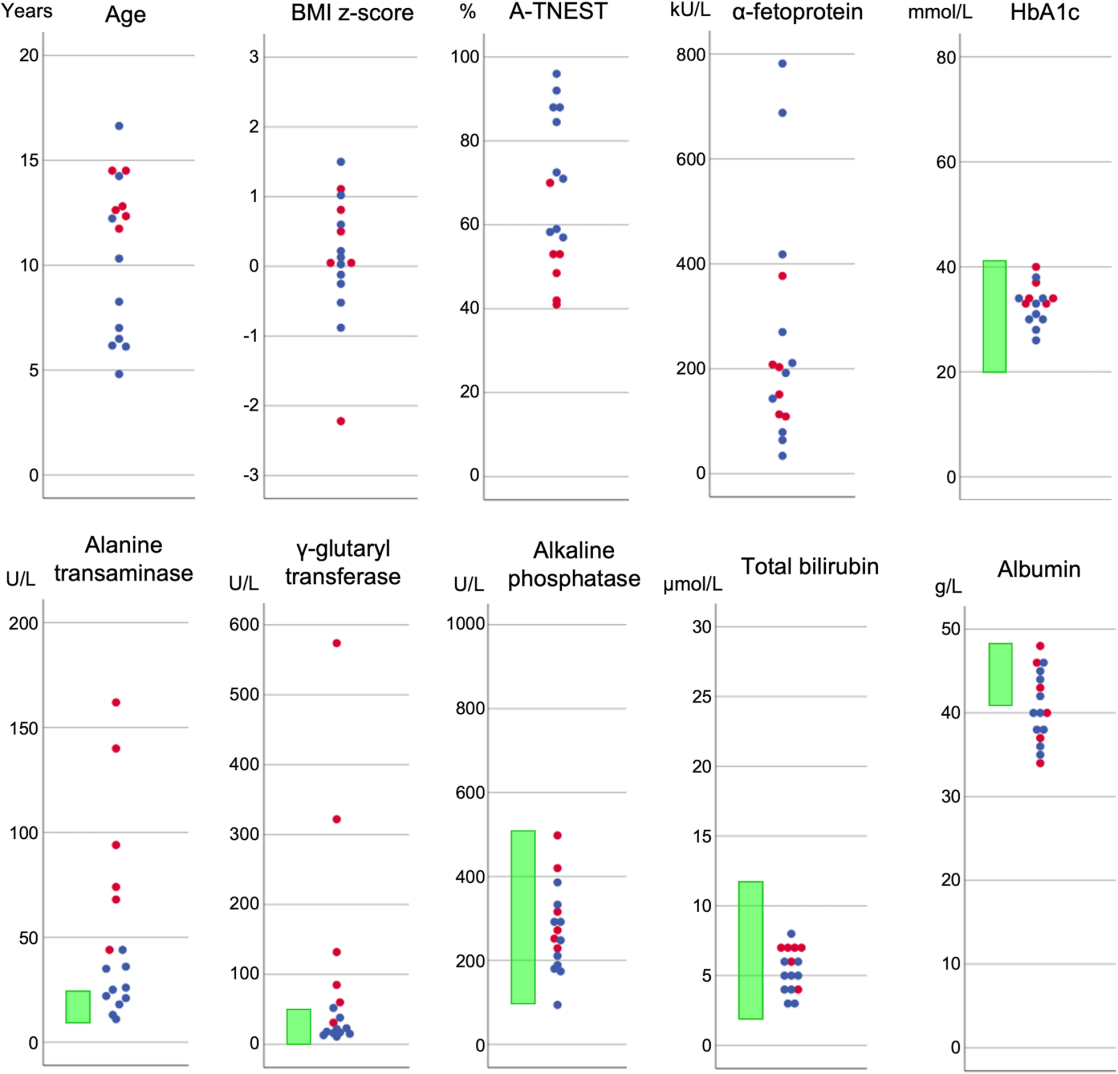
Distribution of laboratory and clinical results for participants who completed the T1 mDIXON MRI scan. Participants with previous diagnosis of liver steatosis are shown as red markers and participants with no previous diagnosis of liver steatosis are shown as blue markers. Laboratory reference ranges are shown as the green range marker (BMI—body mass index, A–TNEST—A–T Neurological Examination Scale Toolkit)

Eighteen children and young people were enrolled, of which 16 completed the whole-body mDIXON acquisition. Participant characteristics are shown in Table 1, and distribution of clinical metrics are shown in Fig. 2. Of these 16 participants, 6 had a prior diagnosis of liver steatosis based on the presence of raised liver enzymes and ultrasonography. Participants with a previous diagnosis of liver steatosis had higher ALT (median 84 U/L, range 44–162 U/L, vs 23.5 U/L, range 11–44 U/L, *p* < 0.001) and GGT (median 108.5 U/L, range 31–574 U/L, vs 17.5 U/L, range 11–52 U/L, *p* < 0.001), were older (median 12.7 years, range 11.7–14.5 years, versus 7.6 years, range 4.8–16.6 years, *p* = 0.042) and had more severe neurological disability (median 50.75%, range 41–70%, versus 78.5%, range 57–96%, *p* = 0.002) compared to participants without a previous diagnosis of liver steatosis. There was no difference in AFP, ALP, total bilirubin, albumin, HbA1c and BMI z-score between participants with and without previous diagnosis of liver steatosis.

**Table 1:** Summary of patients characteristics

Variable		Total n
Sex		16
Male	8 [50%]	
Female	8 [50%]	
Age (years)	12.0 (4.8, 16.6)	16
A–TNEST	64.5 (41, 96)	16
Tube feeding		16
Yes	0 [0%]	
No	16 [100%]	
BMI z-score	0.09 (− 2.22, 1.5)	16
Previous diagnosis of liver steatosis	16	
Yes	6 [37.5%]	
No	10 [60.25%]	
AFP (kU/L)	197.5 (34, 782)	16
ALP (U/L)	262 (94, 498)	16
ALT (U/L)	35.5.(11, 162)	16
GGT (U/L)	27 (11, 574)	16
T-Bil (umol/L)	5.5 (3, 8)	16
Alb (g/L)	40 (34, 48)	16
HbA1c (mmol/mol)	33 (26, 40)	15

*BMI* body mass index, *AFP* alpha-fetoprotein, *ALP* Alkaline phosphatase, *ALT* Alanine transaminase, *GGT* gamma-glutamyl transferase, *T-Bil* total bilirubin, *Alb* Albumin, *HbA1c* haemoglobin A1c, *A–TNEST* A–T Neurological Examination Scale Toolkit

Values are number [percentage] or median (range)

Of these, 5 patients were excluded from further analysis due to movement artefacts significantly degrading the T1-weighted mDIXON image quality (n = 3) or creating the T1-FF image was not possible because the original image of the water image was not correctly archived (n = 2). The three participants whose scans were excluded due to movement artefacts were all 6 years old. Therefore, a total of 11 patients (age, 12.1 ± 1.0 years; range, 4.8–16.6) were finally included in the analysis of T1-FF.

**Fig. 3:**
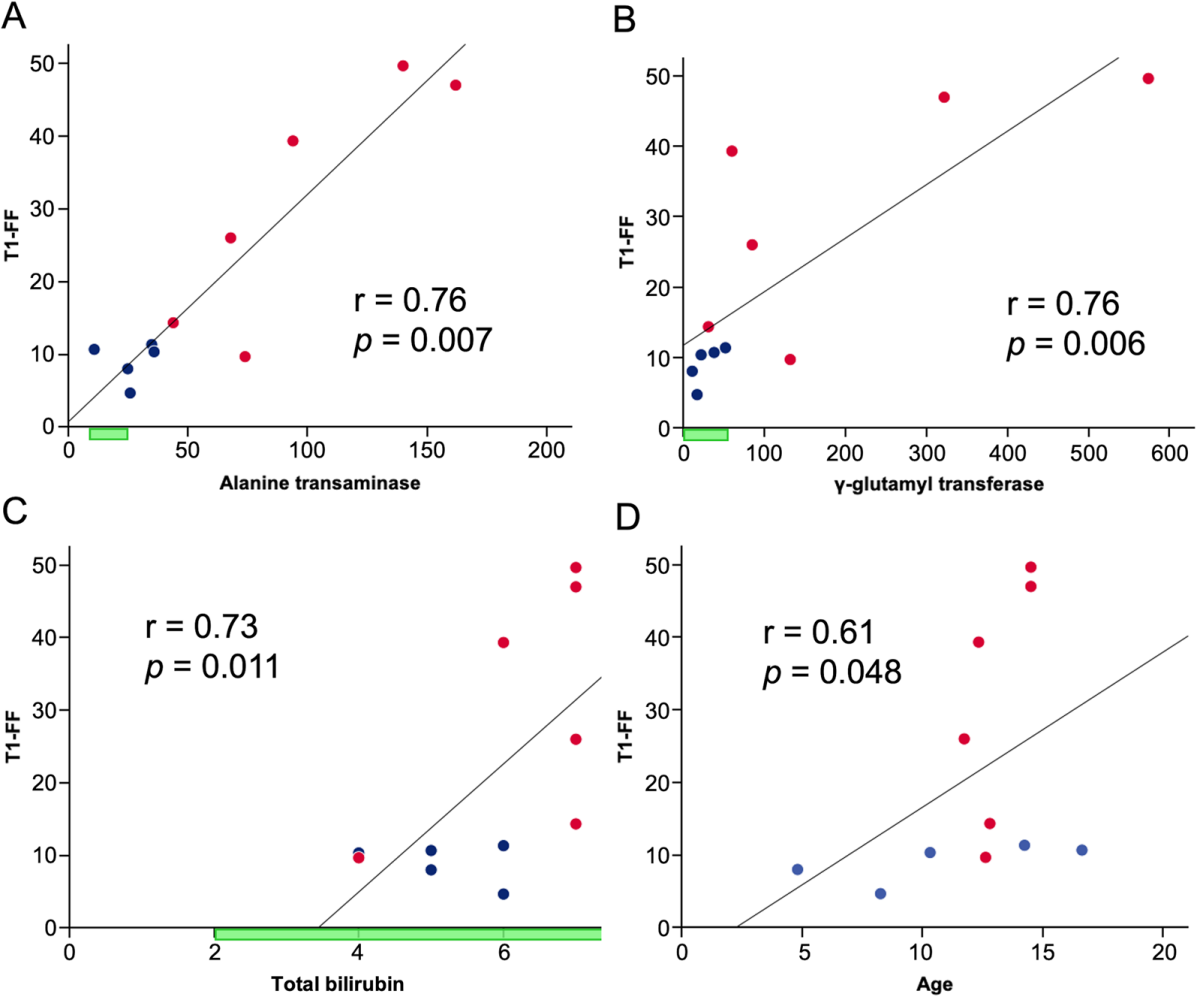
Scatter plot and linear trend line of the T1 weighted mDixon-based fat fraction value (T1-FF) in association with **A** Alanine transaminase, **B** γ-glutamyl transferase, **C** total bilirubin, and **D** age. Participants with previous diagnosis of liver steatosis are shown as red markers and participants with no previous diagnosis of liver steatosis are shown as blue markers. Laboratory reference ranges are shown as the green range marker

The median T1-FF of the liver was 11.3% (range 4.7% to 49.7%). Out of 11 patients, 10 (91%) had T1-FF > 5.56%, in keeping with steatosis. Participants with a previous clinical diagnosis of steatosis had higher T1-FF than those without previous diagnosis of liver steatosis (median 32.7%, range 9.7–49.7%, versus 10.3%, range 4.7–11.3%, *p* = 0.030). Exploratory correlation analysis found that T1-FF correlated with ALT (r = 0.76, *p* = 0.007), GGT (r = 0.76, *p* = 0.006), total bilirubin (r = 0.73, p = 0.011) and age (r = 0.61, *p* = 0.048) (Fig. 3). No statistical differences were observed between T1-FF and any other variables (Table 2).

**Table 2:** Spearman's correlation coefficients between T1-FF and other valuables

	r	p-value
Age	0.61	0.048*
BMI z-score	− 0.06	0.873
AFP	− 0.15	0.670
ALP	0.02	0.958
ALT	0.76	0.007*
GGT	0.76	0.006*
T-Bil	0.73	0.011*
Alb	0.34	0.311
HbA1c	0.32	0.361
A–TNEST	− 0.34	0.311

*T1-FF* T1 weighted mDixon-based fat fraction value, *BMI* body mass index, *AFP* alpha-fetoprotein, *ALP* Alanine transaminase, *ALT* alanine aminotransferase, *GGT* gamma-glutamyl transferase, *T-Bil* Total bilirubin, *Alb* Albumin, *HbA1c* hemoglobin A1c, *A–TNEST* A–T Neurological Examination Scale Toolkit * statistically significant

## Discussion

This study assesses the feasibility of the mDixon MRI for the evaluation of liver steatosis in children with A–T. In 11 out of 16 participants, liver fat-fraction images with sufficient image quality to assess liver fat fraction were obtained. Our findings that participants with known diagnosis of liver steatosis have higher T1-FF than those without, and that T1-FF correlates with markers of liver health (ALT and GGT) provide evidence that the mDIXON approach is effective in identifying the target pathology.

Our study shows that this approach is feasible in children with A–T, a neurodegenerative disease that is associated with involuntary movements and respiratory problems. Movement of children during MRI is a common problem, and given that our study population has the additional issue of disease associated involuntary movements, it is not surprising that we had to discard three datasets due to participant motion. However, it should be noted that our research protocol did not allow the use of sedation or anesthesia which are commonly used in routine clinical practice, and we did not ask participants to breath-hold during the scan due to the respiratory problems experienced by children with A–T. Hence, we feel that discarding of MRI data due to participant motion does not significantly compromise our demonstration of feasibility because in routine clinical use sedation or anesthesia would be an option for minimizing motion and (in the case of general anesthesia) controlling ventilation.

Interestingly, in our study, all three cases excluded due to motion artefact were 6 years old, suggesting that artefact-related image degradation may be influenced not only by neurodegeneration in older children, but also by reduced compliance and inability to remain still in younger participants. These findings highlight the need for age-specific imaging strategies and preparation methods to improve image quality in younger patients, especially in settings where sedation or breath-holding are not feasible.

Moreover, compared to our previous work [18], which focused on whole-body MRI for malignancy screening, the present study applied more stringent image quality criteria. In the earlier cancer screening protocol, a scan was considered successful if it enabled detection of gross lesions such as tumors, and moderate motion artefacts were generally acceptable. In contrast, the current study required high-quality, motion-free T1-weighted mDixon images with correct archiving of both water and fat images in order to generate reliable T1-FF maps. Some datasets were excluded due to missing water images, which are sometimes not archived during routine tumor screening protocols. This methodological difference explains the higher rate of imaging exclusions in our study and should be taken into account when comparing feasibility outcomes between these analyses.

The relevance of liver involvement in people with A–T is likely to increase as life expectancy improves and the impact of other features of the disease are reduced through better supportive care (e.g. improved nutrition, respiratory care, immune support etc.) or innovative disease modifying treatment approaches [28, 29]. Mitochondrial dysfunction and metabolic stress, both of which are well-documented features of A–T [30] are likely to contribute to the development of hepatic steatosis in this population. The authors emphasized that impaired mitochondrial function in A–T leads to increased oxidative stress and dysregulated lipid metabolism, thereby creating a metabolic environment prone to fat accumulation. These alterations may predispose individuals with A–T to hepatic steatosis, even in the absence of conventional risk factors such as obesity or type 2 diabetes. Consequently, there is a need to identify methods and develop protocols for monitoring the development and progression of liver involvement in A–T. Previous research has shown that age is an important risk factor for liver and metabolic abnormalities in patients with A–T [31]. Older individuals tend to exhibit a higher frequency of hepatic and metabolic alterations, supporting the idea that age-related progression plays a key role in liver involvement. A recent study using transient elastography showed that A–T patients over the age of 12 were more likely to have steatosis than those under the age of 12, which is consistent with the results of the current study [12]. However, as illustrated in Fig. 3D, while severe steatosis was more common in older participants, some cases remain with mild steatosis.

The liver enzymes ALT and GGT are known to be associated with liver disease and liver-related mortality and have been used as predictors of liver disease [32–34]. In our study, T1-FF demonstrated a strong positive correlation with both ALT (r = 0.76, p = 0.007) and GGT (r = 0.76, p = 0.006), which is notable given the relatively small sample size (n = 11). While such strong correlations must be interpreted with caution, similar results have been reported in larger studies using controlled attenuation parameter (CAP) derived from transient elastography, supporting the biological plausibility of our findings [12]. Importantly, as shown in Fig. 3A, B, several participants with normal ALT and GGT values already exhibited elevated T1-FF, consistent with early-stage steatosis. This suggests that liver enzyme levels alone may not be sufficient for early detection of steatosis, and that imaging-based fat quantification methods, such as the one used in this study, may offer complementary diagnostic value. While a previous study [12] reported significant correlations between CAP and metabolic parameters such as triglycerides, LDL-HDL ratio, and HbA1c, we did not observe a correlation between T1-FF and HbA1c in our study. A likely explanation is that all participants in our cohort had HbA1c values within the normal range, resulting in limited variability and reduced statistical power to detect associations. These limitations reduce the likelihood of identifying meaningful relationships between glycaemic status and liver fat content. However, the role of metabolic dysfunction in hepatic steatosis is well established, and future longitudinal studies are warranted to evaluate the effects of glycaemic control and diabetes treatment on liver fat accumulation in patients with A–T. In addition, previous research has shown that treatment of type 2 diabetes may reduce hepatic steatosis in patients with A–T, suggesting that improved glycaemic control could play a therapeutic role in managing liver fat accumulation in this population [35]. Although a statistical correlation was observed between the T1-FF and total bilirubin, the relevance of this finding is questionable given that all total bilirubin measurements were within the normal range.

Liver biopsy remains the gold standard for diagnosing the severity of liver steatosis, the presence of nonalcoholic steatohepatitis (NASH), and the stage of fibrosis. However, there are several limitations and complications associated with this procedure, including sampling error, invasiveness, severe bleeding, and the risk of pneumothorax [36]. Furthermore, the risks associated with liver biopsy in A–T patients are greater than in the general pediatric population, due to the presence of pulmonary disease and the inherent risks associated with anesthesia [37]. Therefore, a biopsy is not the ideal method for diagnosing or monitoring liver disease in patients with A–T, particularly given the necessity for repeated sampling to observe changes over time.

Non-invasive imaging tools have been employed for the purpose of qualifying and quantifying hepatic fat content, including ultrasound, computed tomography (CT), and MRI. Although liver ultrasound is the most commonly used imaging modality for the assessment of hepatic steatosis, the conventional technique is only able to detect steatosis when more than 30% of the hepatocytes are affected by steatosis [38]. Furthermore, a systematic review has determined that liver ultrasound is not an accurate method for grading steatosis, with a positive predictive value for detecting steatosis of only 62–77% [39, 40]. Transient elastography using CAP has emerged as a reliable and practical method for assessing hepatic steatosis. Recent studies have demonstrated its high diagnostic accuracy, especially for early-stage steatosis [41]. CAP offers significant advantages over MRI in terms of portability, repeatability, cost-effectiveness, and point-of-care availability. These features make CAP particularly useful in longitudinal or interventional studies. While MRI provides superior spatial information and anatomical resolution, it is less suitable for frequent follow-up assessments. Therefore, CAP and MRI should be considered complementary tools for the evaluation and monitoring of liver involvement in A–T. CT is a widely available imaging modality that can provide rapid volumetric imaging and an objective, quantitative assessment of hepatic X-ray attenuation, which is related to liver fat content. However, it is generally not recommended in children because of the radiation exposure, especially in A–T patients who have significantly increased sensitivity to radiation.

When performed optimally, magnetic resonance spectroscopy (MRS) shows remarkable diagnostic efficacy for liver-related applications, with a high degree of concordance with histological assessments [42]. However, MRS has important limitations that prevent its widespread use in clinical and research settings. It is time-consuming to perform and requires expertise to analyze. It is generally only available in academic centers. Furthermore, sampling error is difficult to avoid because of its limited spatial coverage. Therefore, when using MRS to monitor the progress of patients, it may be difficult to reproduce the exact sampling position, which is problematic for longitudinal monitoring.

This study employed one of the simplest implementations of the mDixon method, the T1-weighted two-point mDixon method. This method involves acquiring images at specific echo times when water and fat signals are added and subtracted, respectively, resulting in the generation of"in-phase"and"opposed-phase"images [16]. The mDixon-based imaging is regarded as a reliable method for quantifying liver steatosis, as evidenced by the multiple studies that have employed mDixon-based techniques to quantify liver fat content with MRS as the reference standard [17, 25, 43]. These studies have demonstrated that mDixon-based imaging is as accurate as MRS and exhibits a strong correlation with MRS, as well as with histology. Although these studies used more complex techniques based on chemical shift, such as proton density fat fraction or multipoint Dixon, to provide accurate measurement of liver fat, it should be noted that T1-weighted two-point mDixon sequence which we used in our study is available on all scanners and is usually included in all upper abdominal MR protocols. This is the first study to use T1-weighted mDixon MRI to assess the degree of steatosis in A–T patients and shows that T1-FF can be measured in correlation with hepatobiliary enzymes such as ALT and GGT, even in pediatric A–T patients who are difficult to obtain images of under ideal conditions due to their underlying disease and young age.

Our study has several limitations. Firstly, this study lacked a control group and did not include liver ultrasound or blood test data obtained at the same time as the MRI. These limitations, inherent to the post-hoc design, underscore the exploratory nature of this analysis. Nonetheless, the insights gained from this feasibility study may help guide the design of future prospective investigations incorporating synchronized multimodal assessments. Secondly, we used ROIs that were drawn manually in each of the liver lobes, rather than covering the whole liver. Some previous studies have reported that the fat fraction values obtained for the whole liver were slightly higher than those obtained for the manually drawn ROI [25, 44]. This may be due to including periportal fat and fat in the intrahepatic fissure when segmenting the whole liver. It is reassuring to note that a strong positive correlation was previously reported between MRS and multi-echo Dixon with manually placed ROIs (r = 0.988, r^2^ = 0.978, p < 0.001) [25]. Additionally, as movement artefacts occurring in children with A–T may blur the liver margin, we thought that ROIs placed within the parenchyma away from edges would be less likely to have partial volume inaccuracies. Thirdly, the calculated T1-FF might be systematically overestimated due to liver fat signal T1 effects [45]. However, when assessing liver steatosis in A–T patients in a clinical setting, the important thing is to capture changes over time, so we consider that this characteristic will not be a major problem in clinical use. Fourthly, this study did not include CAP measurements, which are considered the current non-invasive gold standard for assessing steatosis and fibrosis. Future studies should consider combining MRI with CAP to leverage the complementary strengths of both modalities—MRI’s anatomical precision and CAP’s point-of-care repeatability—to enhance clinical translatability. Finally, this study included a relatively small number of patients, and the findings need to be confirmed in a larger cohort.

In conclusion, T1-weighted mDixon MRI is feasible for detecting steatosis in children with A–T and demonstrates a higher prevalence of liver steatosis than previously reported in paediatric A–T cohorts, including children with normal ALT and GGT. MRI quantified liver T1-FF correlates with markers of liver health, and further evaluation in a larger cohort is warranted to establish the role of MRI in monitoring the development of liver steatosis in children with A–T in clinical practice.

## Data Availability

Due to privacy concerns and to protect patient confidentiality, the imaging data that support the findings of this study are not publicly available. Access to the data may be granted under strict confidentiality agreements upon request. For inquiries related to data access, please contact the corresponding author.
